# Computational Study of pH-sensitive Hydrogel-based Microfluidic Flow Controllers

**DOI:** 10.3390/jfb2030195

**Published:** 2011-08-25

**Authors:** Jundika C. Kurnia, Erik Birgersson, Arun S. Mujumdar

**Affiliations:** 1 Department of Mechanical Engineering, National University of Singapore, 9 Engineering Drive 1, 117576 Singapore; E-Mail: jc.kurnia@nus.edu.sg; 2 Department of Chemical and Bio-Molecular Engineering, National University of Singapore, 5 Engineering Drive 2, 117576 Singapore; 3 Mineral, Metal and Material Technology Centre, National University of Singapore, 9 Engineering Drive 1,117576 Singapore; E-Mail: mpeasm@nus.edu.sg

**Keywords:** flow control, hydrogel, mathematical model, microchannel, microfluidic, pH, simulation

## Abstract

This computational study investigates the sensing and actuating behavior of a pH-sensitive hydrogel-based microfluidic flow controller. This hydrogel-based flow controller has inherent advantage in its unique stimuli-sensitive properties, removing the need for an external power supply. The predicted swelling behavior the hydrogel is validated with steady-state and transient experiments. We then demonstrate how the model is implemented to study the sensing and actuating behavior of hydrogels for different microfluidic flow channel/hydrogel configurations: e.g., for flow in a T-junction with single and multiple hydrogels. In short, the results suggest that the response of the hydrogel-based flow controller is slow. Therefore, two strategies to improve the response rate of the hydrogels are proposed and demonstrated. Finally, we highlight that the model can be extended to include other stimuli-responsive hydrogels such as thermo-, electric-, and glucose-sensitive hydrogels.

## 1. Introduction

As a relatively new branch of science and technology, microfluidics, which emerged in the early 1990s [1,2,3], has attracted much attention for its diverse applications, ranging from ink-jet printers and fuel injection [4], over surface processing and biological assay [5], to control system, heat management and display technology [6]. Other fields where microfluidic systems are considered and employed include micromixing [7,8], biology and biochemical analysis [9,10,11,12]. For several of these applications, the ability to manipulate the fluid flow within the microchannels is essential [12]; therefore, considerable effort has been devoted to develop microfluidic flow controllers. The majority of flow controllers in microfluidics systems are miniaturized version of their conventional macroscale counterparts [13], which are generally integrated devices comprising electrical, mechanical and optical elements with individual functions. These conventional microfluidic flow controllers have two major drawbacks: the inherent difficulty in assembling the various components into a single system and the requirement of an external power supply, both of which limit their implementation in numerous applications [14,15].

In contrast to conventional microfluidic flow controllers, stimuli-sensitive hydrogels can be employed without external power supply. Moreover, hydrogels offer significant reduction in the complexity of a microsystem due to their unique stimuli-sensitive ability; that is, stimuli-sensitive hydrogels can sense changes in its environment—temperature, pH, glucose, electric field and pressure—and then swell or shrink correspondingly [16,17]. During swelling, certain hydrogels are able to absorb large amounts of water leading to a large swelling ratio [18,19,20]. Hydrogels could therefore replace the major components in microfluidics flow controllers such as sensors, signal processors, regulators and actuators [21]. Moreover, their high water content and soft consistencies lend them excellent biocompatibility, allowing application of this hydrogel-based system in biomedical and biotechnical fields. Due to their potential for active flow control, numerous experimental designs and studies of hydrogels as flow controller in microfluidic systems have been conducted [15,21,22,23,24,25,26,27]. In contrast, few studies concerning mathematical modeling and simulation have been reported [28,29,30]; hence, it is of interest to develop mathematical models which can aid in the synthesis and design of hydrogels as microfluidic flow controllers.

**Figure 1:**
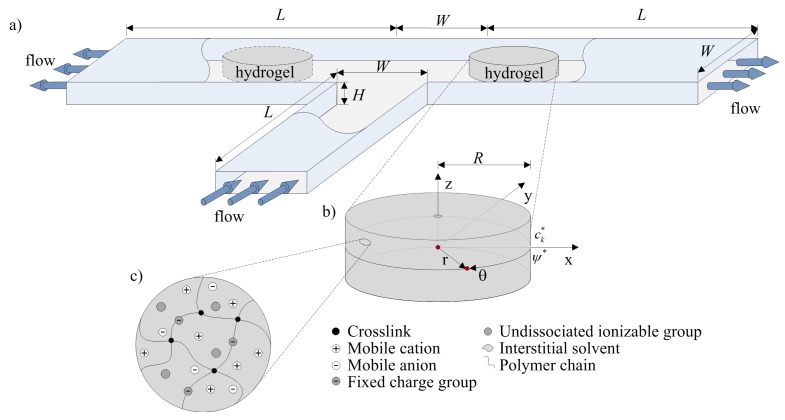
Schematic representation of (**a**) a flow configuration with hydrogels act as autonomous valves; (**b**) an axially constrained hydrogel subject to alteration in pH; and (**c**) a hydrogel.

This paper addresses the sensing and actuating behavior of hydrogels as microfluidic flow controller with the aim to derive and analyze a simple mathematical model for a pH-sensitive hydrogel that can be integrated with the external flow in a microfluidic flow system; e.g., in a T-junction, as illustrated in Figure 1. The mathematical model, which takes into account conservation of momentum, mass and ions for laminar incompressible flow and the sensing/deformation of a pH-sensitive hydrogel, is derived, analyzed and presented in Section 2. Details of the numerical procedure are outlined in Section 3. Calibration and validation with steady-state and transient experiments [31] is then carried out for the deformation of the hydrogel as a function of pH, after which we demonstrate how the model can be employed to study the sensing and actuating behavior of the hydrogel as microfluidic flow controller. The flow configurations considered in this paper are (*i*) a T-junction with hydrogels in one branch and (*ii*) a T-junction with hydrogels in each branch; the latter has two hydrogels with opposite behavior: a positive pH-responsive hydrogel, which swells as the pH increases, and a negative pH-responsive hydrogel, which shrinks as the pH increases. We finish with conclusions, in which we highlight how the model can be generalized for other types of stimuli-responsive hydrogels.

## 2. Mathematical Formulation

In this section, we derive a mathematical model that incorporates the conservation of momentum, mass and species for a laminar incompressible flow as well as the sensing and deformation of the hydrogel. The hydrogel considered in this paper is pHEMA (polyhydroxylethylmethacrylate), which is a pH-sensitive hydrogel [31]. The hydrogel is embedded as microfluidic flow controller in a microchannel (see Figure 1); as the hydrogel shrinks and swells depending on the pH of the solution in the system (sensing), it affects (controls) the overall flow in the system. The solution is aqueous with protons (H^+^), sodium ions (Na^+^), hydroxide ions (OH^−^), and chloride ions (Cl^−^) at ambient temperature.

### 2.1. Governing Equations

For laminar, incompressible flow inside the microchannel, conservation of mass and momentum are given by the Navier–Stokes equation:

(1) $$\nabla \cdot {\text{v}}^{\left(\text{f}\right)}=0,$$

(2) $$\rho_{0}^{\left(f\right)}\frac{\partial {\text{v}}^{\left(f\right)}}{\partial t}+\rho_{0}^{\left(f\right)}\left({\text{v}}^{\left(f\right)}\cdot \nabla \right){\text{v}}^{\left(f\right)}=-\nabla p+\mu^{\left(f\right)}\nabla^{2}{\text{v}}^{\left(f\right)}$$

where $\rho_{0}^{\left(f\right)}$ is the true density of fluid, *μ*^(*f*)^ is the dynamic viscosity of the fluid, *p* is the fluid pressure and **v**^(*f*)^ is the fluid velocity.

We account for ion transfer inside the channel and the hydrogel with the Nernst–Planck equation and electroneutrality condition [32,33,34], which can be expressed as

(3) $$\frac{\partial c_{k}}{\partial t}+\nabla \cdot \left(c_{k}{\text{v}}^{\left(f\right)}\right)=D_{k}\nabla^{2}c_{k}+\frac{z_{k}{\text{FD}}_{k}}{ℜT}\nabla \left(c_{k}\nabla \psi \right)$$

(4) $$z_{f}c_{f}+\sum_{k}^{N}z_{k}c_{k}=0$$

where *F* is Faraday's constant, ℜ is the universal gas constant, *ψ* is the electric potential, *D_k_* is the diffusive coefficient, *z_k_* and *c_k_* are valence and concentration of the ion species *k* (= H^+^, Na^+^, OH^−^and Cl^−^) respectively; *ϵ*_0_ is the permittivity for vacuum, *ϵ* is the dielectric constant of medium relative to vacuum, *z_f_* is the valence of fixed charge and *c_f_* is the fixed charge concentration inside the hydrogel.

For the hydrogel, conservation of mass is solved for a biphasic mixture comprising the solid and fluid phase, whereas conservation of momentum for the hydrogel is considered in terms of Navier's equation with infinitesimal deformations and generalized Darcy's law for a moving porous medium [35,36,37]; that is

(5) $$\nabla \cdot {\text{v}}^{\left(\text{p}\right)}=-\frac{1}{\rho_{0}^{\left(f\right)}}\nabla \cdot \text{q}$$

(6) $$\nabla p=-\frac{\mu^{\left(f\right)}}{\kappa^{\left(p\right)}\rho_{0}^{\left(f\right)}}\text{q}$$

(7) ∇⋅σ=0

where **q** =*ρ*^(*f*)^**v**^(*r*)^ is the Eulerian relative flow vector of the fluid phase with respect to the polymer phase, **v**^(*r*)^ = **v**^(*f*)^ − **v**^(*p*)^ is the velocity of fluid relative to the solid phase velocity, **v**^(*i*)^ is the intrinsic velocity of phase *i* (solid and fluid), *κ*^(*p*)^is the permeability of polymer phase, *σ* is the mixture stress tensor, and $\rho^{\left(i\right)}=\phi^{\left(i\right)}\rho_{0}^{\left(i\right)}$ is the density of phase *i*, where $\rho_{0}^{\left(i\right)}$ is the true density of each phase. The velocity of the solid phase can be defined as the rate of deformation; that is, **v**^(*p*)^ = ∂**u**^(*p*)^ /*∂t*, where **u** is the deformation of the hydrogel.

### 2.2. Constitutive Relations

The mixture stress tensor for the hydrogel is given by [35]

(8) $$\sigma =-p\text{I}+\sigma_{\text{eff}}^{\left(p\right)}$$

where $\sigma_{\text{eff}}^{\left(p\right)}$ is the elastic stress tensor of the polymer phase in the hydrogel. We treat the polymer phase as an isotropic elastic material, whence the elastic stress tensor [32,38] of the polymer phase can be expressed as

(9) $${\text{σ}}_{\text{eff}}^{\left(p\right)}=\lambda_{s}\left(\text{E}:\text{I}\right)\text{I}+2\mu_{s}\text{E}$$

here, the Lamé coefficients, *λ_s_* and *μ_s_* and the elastic strain tensor of the solid phase, **E** are defined as [39] respectively; *E*_0_ is Young's modulus and *ν* is the Poisson ratio.

(10) $$\lambda_{s}=\frac{\nu E_{0}}{\left(1+\nu \right)\left(1-2\nu \right)}$$

(11) $$\mu_{s}=\frac{E_{0}}{2\left(1+\nu \right)}$$

(12) $$\text{E}=\frac{1}{2}\left[\nabla \text{u}+{\left(\nabla \text{u}\right)}^{T}\right]$$

The osmotic pressure inside the hydrogel comprises the mixing and ionic contributions, which can be expressed as

(13) $$p^{\left(\text{osm}\right)}=\underset{\text{mixing}}{\underset{︸}{-\frac{k_{B}T}{V_{m}}\left(\phi^{\left(p\right)}+\chi \phi^{{\left(p\right)}^{2}}+\ln\left(1-\phi^{\left(p\right)}\right)\right)}}+\underset{\text{ionic}}{\underset{︸}{ℜT\sum_{k}^{N}\left(c_{k}-c_{k}^{*}\right)}}$$

where *k_B_* is Boltzmann's constant, *V_m_* is the equivalent volume occupied by one monomer, *N_x_* is the degree of polymerization, *χ* is the polymer-solvent interaction parameter, and $c_{k}^{*}$ is the concentration in the fluid channel (outside hydrogels) of the ion species *k*. The polymer-solvent interaction parameter, *χ*(*T*, *ϕ*^(*p*)^), is generally expressed as a function of temperature and polymer volume fraction [40,41,42]; *i.e.*,

(14) $$\chi =-\frac{\Delta s}{k_{B}}+\frac{\Delta h}{k_{B}T}+\chi 2\phi^{\left(p\right)}$$

where Δ*h* and Δ*s* denote the changes in enthalpy and entropy, and *χ*_2_ is a parameter to express the polymer volume fraction dependence of the interaction parameter.

The fixed charge concentration is given by [31]

(15) $$c_{f}=\{\begin{array}{cc} \frac{1}{ℌ}\frac{c_{f}^{0}K_{a}}{\left(K_{a}+c_{\text{H}+}\right)}, & \text{inside the hydrogel},\\ 0, & \text{outside the hydrogel} \end{array}$$

where $c_{f}^{0}$ and *c*_H+_ are the initial fixed charge and hydrogen ion concentrations, respectively, *K_a_* is the dissociation constant of the fixed charge group and ℌ is the hydration state of the hydrogel, which is defined as the ratio of the volume of the fluid phase to the volume of the polymer phase inside the hydrogel, ℌ = *V_f_*/*V*_0_. For axially restrained cylindrical hydrogels, hydration can be related to the strain of hydrogel as

(16) $$ℌ=E_{rr}+E_{\theta \theta }$$

where *E_rr_* and *E_θθ_* are the radial and tangential strains, given by [43]

(17) $$E_{rr}=\frac{\partial u_{r}}{\partial r}$$

(18) $$E_{\theta \theta }=\frac{u_{r}}{r}$$

respectively Note that—compared to De *et al* [31]—an additional term for hydration has been included: *viz.*, tangential strain, since the deformation in the radial direction will trigger strain in the tangential direction [44]; hence,hydration should take into account total strain in radial and tangential direction.

The dynamic viscosity of the fluid phase can be expressed as [35]

(19) $$\mu^{\left(f\right)}=a_{1}{\left(T+a_{2}\right)}^{a_{3}}$$

and the permeability of the polymer network is given by [45]

(20) $$\kappa^{\left(p\right)}=\kappa_{0}^{\left(p\right)}{\left(\frac{\phi^{\left(f\right)}}{\phi^{\left(p\right)}}\right)}^{n}$$

where 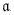
*_i_*, $\kappa_{0}^{\left(p\right)}$ and 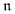 are constants summarized in Table 1.

The effective diffusivity of ion inside the hydrogel is taken into account by Bruggeman equation [46]:

(21) $$D_{k}^{\left(\text{eff}\right)}={\left(\phi^{\left(f\right)}\right)}^{3/2}D_{k}$$

where *D_k_* is the diffusive coefficient of ion species in water.

Here, pH and pKa are the negative logarithm of hydrogen ion concentration and dissociation constant given by

(22) $$pH=-\log_{10}\left(c_{1}\times c_{\text{H}+}\right)$$

(23) $$pK_{a}=-\log_{10}\left(c_{1}\times K_{a}\right)$$

respectively; 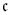
_1_ is a constant presented in Table 1.

### 2.3. Boundary and Initial Conditions

The boundary conditions can be summarized as follows:

- At the inlet of the channel, we prescribe
  (24) $$c_{k}=c_{k,\text{in}},\psi =\psi_{\mathrm{in}},p=p_{\text{in}}$$
- At the outlet of the channel, we prescribe
  (25) $$\nabla c_{k}\cdot \text{n}=\nabla \psi \cdot \text{n}=0,p=p_{\text{out}}$$
- At the walls of the channel, we prescribe
  (26) $$\nabla c_{k}\cdot \text{n}=\nabla \psi \cdot \text{n}=0,{\text{v}}^{\left(f\right)}=0$$
- In the centre of hydrogel, we prescribe
  (27) u=0
- At the hydrogels/fluid interface, the fluid velocity and fluid pressure are prescribed as
  (28) $${\text{v}}^{\left(f\right)}{|}_{-}={\text{v}}^{\left(f\right)}{|}_{+},p{|}_{-}=p{|}_{+}+p^{\left(\text{osm}\right)}$$

Initial conditions invoked are

(29) $$c_{k}=c_{k,0},\psi =\psi_{0},p=p_{\mathrm{out}},\text{u}=c_{2}\text{x}$$

Here, **n** is a unit vector normal to the given surface, |_−_ and |_+_ denote condition inside and outside the hydrogel, and 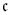
_2_ is a constant presented in Table 1. The boundary condition in the centre of hydrogel is necessary in order to prevent translational movement of the hydrogel and corresponds to the way the hydrogel is attached to the flow channel; see e.g., [14,15].

**Table 1:** Base-case parameters.

**Parameter**	**Value**	**Unit**	**Reference**
*E_0_*	$\{\begin{array}{cc} 0.29 & \text{for}\;\text{pH}<5.5\\ \left(-0.03\text{pH}+0.455\right) & \text{for}\;5.5<\text{pH}<7.5\\ 0.23 & \text{for}\;\text{pH}>7.5 \end{array}$	MPa	[31]
*ν*	0.409	-	[31]
*k_B_*	1 38054 × 10^−23^	JK^−1^	[35]
*V_m_*	3.3 × 10^−28^	m^3^	[35]
*F*	9.648 × 10^4^	C mol^−1^	[32]
ℜ	8.314	JK^−1^ mol^−1^	[32]
*κ*_0_	2 8 × 10^−21^	m^2^	calibrated
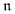	^−^2.5	-	-
*K*_a_	10^−2^	mol m^−3^	[31]
$c_{f}^{0}$	1800	mol m^−3^	[31]
$c_{{\text{Na}}^{+}}^{*}$	300	mol m^−3^	[31]
ψ*	0	V	prescribed
*D_H_*_+_	9.311 × 10^−9^	m^2^ s^−1^	[47]
*D*_Na+_	1.334 × 10^−9^	m^2^ s^−1^	[47]
*D*_Cl−_	2.032 × 10^−9^	m^2^ s^−1^	[47]
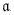 _1_	0.6612	kg m^−1^ s^−1^ K^1.562^	[48]
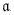 _2_	−229	K	[48]
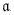 _3_	−1.562	-	[48]
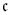 _1_	10^−3^	m^3^ mol^−1^	-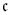 _2_	$\{\begin{array}{cc} 0.15 & \text{for}\;\text{pH}=3\\ 0.93 & \text{for}\;\text{pH}=6\\ 1.12 & \text{for}\;\text{pH}=7 \end{array}$	-	equilibrium model
$\rho_{0}^{\left(f\right)}$	10^3^	kg m^−3^	[49]
Δ*h*	−1 38 ×10^−20^	J	calibrated
Δ*s*	−4.8 × 10^−23^	J K^−1^	calibrated
*χ*_2_	1.34	-	calibrated
*p_in_*	0.02	Pa	-
*p_out_*	0.00	Pa	-
*T*	298	K	-
*L*	1.5 × 10^−3^	m	-
*W*	6.0 × 10^−4^	m	-
*H*	1.8 × 10^−4^	m	-

## 3. Numerical Methodology

The mathematical model is solved with the commercial finite-element solver, Comsol Multiphysics 3.5a. Two geometries—hydrogels and channel—are solved simultaneously. Overall, the mathematical model for the hydrogels and flow inside the channel consist of eight dependent variables: *u_r_*, *c*_1_, *c*_2_, *c*_3_, *u*^(*f*)^, *v*^(*f*)^, *w*^(*f*)^, and *p*^(*f*)^. The geometries are resolved with around 1600–1800 elements to ensure mesh-independent solutions, amounting to around 2.6 × 10^4^–4.4 × 10^4^ degrees of freedom; a finer mesh is chosen at the interface between a hydrogel and the surrounding fluid in the microchannel. The computations were carried out on a computer with a 2.66 GHz dual processor and 4 GB RAM and took around 10–30 min.

## 4. Results and Discussions

### 4.1. Calibration and Validation of the Hydrogel Model

**Figure 2:**
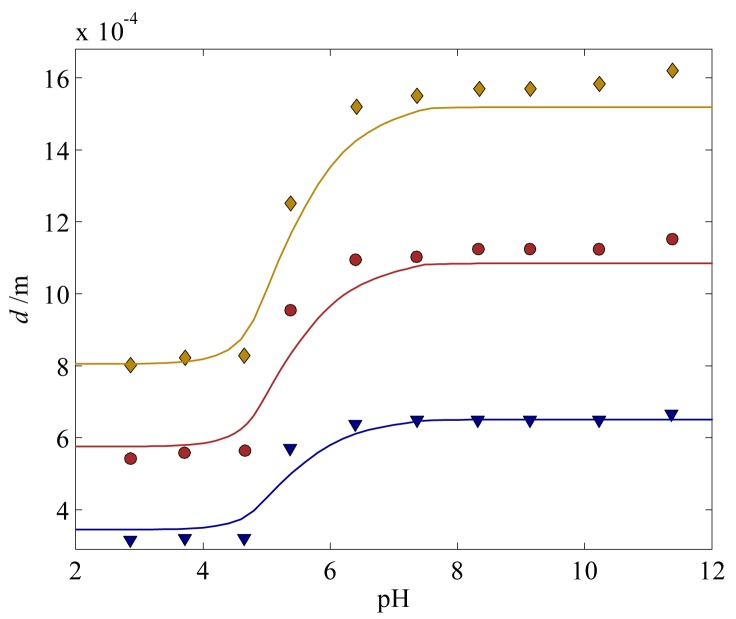
Diameters of hydrogels with respect to pH. The experimentally measured values are [31] ( 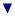) 300 *μ*m, ( 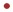) 500 *μ*m, and ( 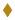) 700 *μ*m. The solids lines are the corresponding model predictions.

Before we study the behavior of a hydrogel and its effect on the overall fluid flow in a microfluidic T-junction, we calibrate Δ*h*, Δ*s*, and *χ*_2_ with the steady-state swelling curve for a diameter of 300 *μ*m (training set) from the experiments by De *et al.* [31], as shown in Figure 2, and validate the deformation of the pH-sensitive hydrogel HEMA with experimental hydrogels with a diameter of 500 and 700 *μ*m (test set). Overall, good agreement is achieved between the model prediction and the experiments. Clearly, the pHEMA hydrogel collapses at low pH and swells at high pH: This pH-induced swelling behavior can be attributed to the presence of acidic groups bound to the polymer chains, which become highly ionized at certain pH value [16,50]. The acidic group inside the hydrogel is only slightly ionized when the pH drops below the pKa of the hydrogel—in this case the pKa is 5. As a result, swelling of the hydrogel at pH changes below pKa is marginal, which is mirrored by the slight increase in the hydrogel radius for *pH* ≲ 4 in Figure 2. As the pH increases and approaches the pKa value, the acidic functional group becomes near to fully ionized by deprotonation, which results in an increase in the fixed charge density, *c_f_* The increase in the fixed charge density, in turn, appears in the osmotic pressure (driving force) and causes a swelling, as depicted in Figure 2 for the pH range 4–6. When the ionization process reaches its saturation point, an increase in pH does not affect swelling behavior of the pHEMA hydrogel [51]. This can be observed in Figure 2 where the hydrogel stops swelling at leading order when pH > 7.

**Figure 3:**
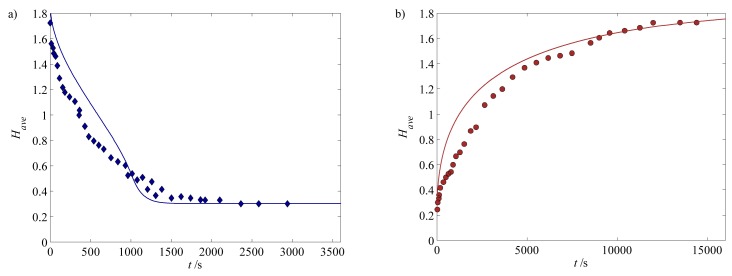
(**a**) Shrinking and (**b**) swelling kinetics for a 300 *μ*m hydrogel for pH change between 3 and 6. The experimentally measured values are ( 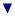) for shrinking and ( 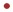)swelling [31,52].

Turning our attention towards the deformation kinetics of the hydrogel, we first calibrate the permeability constant, *k*_0_, for the shrinking of a 300 *μ*m hydrogel when subjected to pH changes from 6 to 3 (training set), as illustrated in Figure 3a. We then validate the deformation kinetics with the corresponding swelling (see Figure 3b) with reasonably good agreement. Overall, we note that the shrinking is approximately ten times faster than the swelling: shrinking and swelling require around 1500 s and 18,000 s respectively in order to reach the new steady state.

Aside from calibration and validation purposes, it is of interest to study equilibrium swelling behaviorand deformation kinetics of a hydrogel since these are two important factors in designing hydrogels for microfluidic flow control: From the equilibrium swelling behaviour, we can identify and modify the properties of hydrogels that affect the swelling ratio and synthesize a hydrogel with the desired swelling ratio for flow control purposes; and from the deformation kinetic behaviour, we cam estimate the response time of the hydrogel when it is employed as microfluidic flow controller, after which we can design or synthesize a hydrogel with a sufficiently fast response for any given microfluidic flow control system.

### 4.2. Flow Behavior inside a T-Junction with one or Several Hydrogels in One Branch

**Figure 4:**
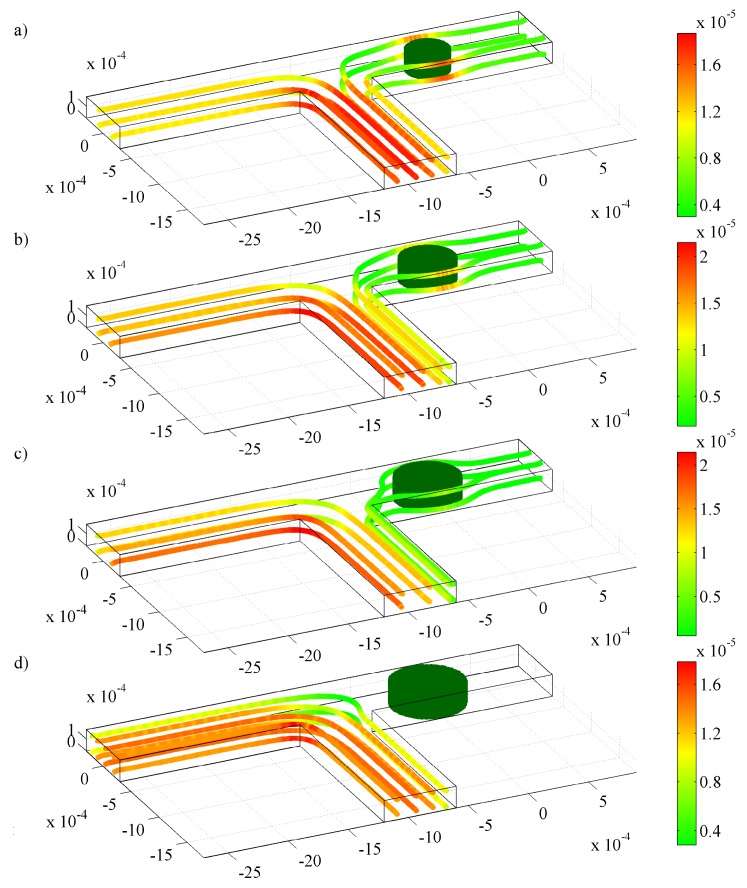
Streamlines and velocity distribution (*m s*^−1^) for laminar flow in a T-junction with one 300 *μ*m hydrogel for pH changes between 3 and 7 at (**a**) *t* = 0 min; (**b**) *t* = 10 min; (**c**) *t* = 50 min; and (d) *t* = 200 min.

**Figure 5:**
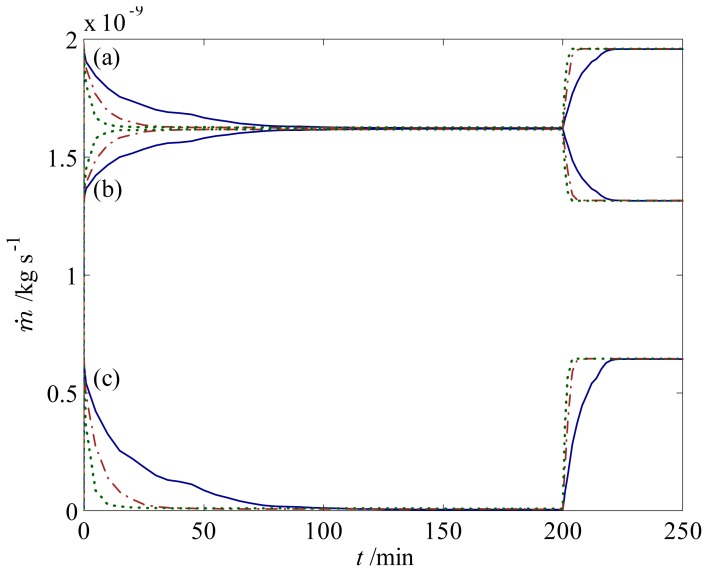
Response of the fluid flow during swelling and shrinking in a T-junction with ( 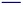) one 300 *μ*m hydrogel, ( 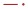) two 150 *μ*m hydrogels, and ( 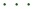) three 100 *μ*m hydrogels. Mass flow rates of the fluid are for pH changes between 3 and 7 at (**a**) the inlet; (**b**) the left outlet; and (**c**) the right outlet.

We proceed further by examining the sensing and actuating behavior of a 300 *μ*m hydrogel in a T-junction when the solution pH is changed between 3 and 7, as illustrated in Figure 4. This configuration represents a simple microfluidic flow controller based on a stimuli-responsive hydrogel—also commonly referred to as resistance-based flow control [15]. Initially, at low pH, the hydrogel is in its shrunken state and thus allows fluid flow (Figure 4a) between itself and the walls of the microchannel. A step change in pH from 3 to 7 is then applied to the system, for which the hydrogel starts to swell towards the new equilibrium and block the channel, as depicted in Figure 4b–d. The mass flow rate of the fluid at the inlet and outlets of the channel as the hydrogel deforms is presented in Figure 5. When the hydrogels reach new equilibrium at *t* ∼ 200 min, a step change in *pH* from 7 to 3 is applied; thus, the hydrogel starts to shrink towards the initial condition. Here, several features are apparent: First, the mass flow rate at the inlet of the channel decrease as the hydrogel swells, similar to that at the left outlet; second, the response of the hydrogel is rather slow—it takes around 120 min for the hydrogel to fully close the channel—which can defeat the purpose of flow control. The reason for the first observation is simple: as the hydrogel swells, it obstructs the flow and creates high resistance for the fluid to flow because of its low permeability. The second observation suggests that we should modify the hydrogel microfluidic system to obtain higher response rates.

In light of the second observation, we demonstrate two strategies in improving a hydrogel's response rate: first, by replacing a single larger hydrogel with multiple smaller hydrogels, and second, by employing a hydrogel with higher permeability (macroporous hydrogel).

**Figure 6:**
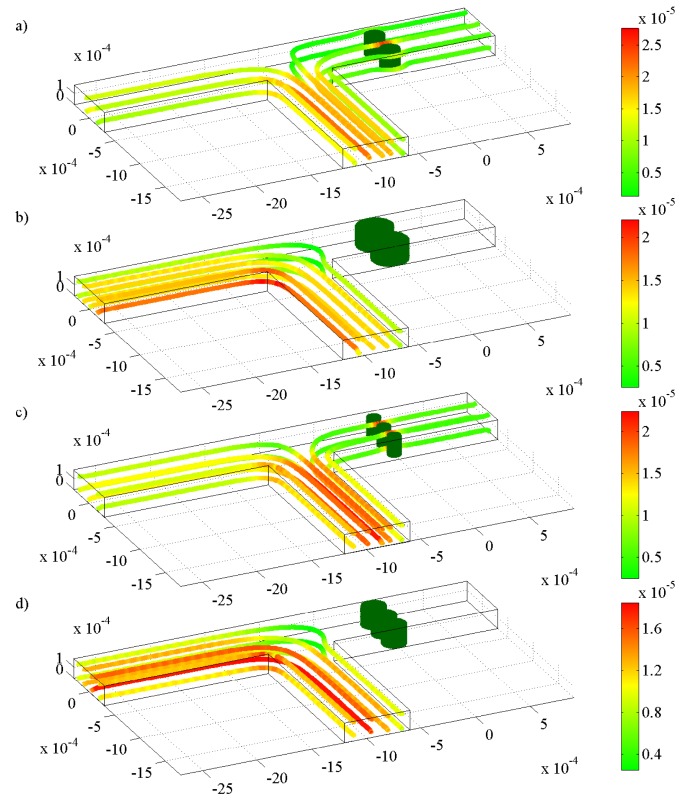
Streamlines and velocity distribution (m s^−1^) for laminar flow in a T-junction with (**a**) two 150 *μ*m hydrogels in a shrunken state; (**b**) two 150 *μ*m hydrogels in a swollen state; (**c**) three 100 *μ*m hydrogels in a shrunken state, and d) three 100 *μ*m hydrogels in a swollen state.

For the first strategy, we implement two hydrogels with the size of 150 *μ*m and three hydrogels with the size of 100 *μ*m to replace the 300 *μ*m, as shown in Figure 6. In doing so, we find that the response time is approximately 3 times faster (for 150 *μ*m hydrogels) and 6 times faster (for 100 *μ*m hydrogels) compared to the corresponding case with a single 300 *μ*m hydrogel, as depicted in Figure 5. The reason for this response time enhancement is the fact that by reducing the size of hydrogel, we shorten the diffusion path of the penetrating fluid, which, in turn, leads to a faster response by the hydrogel. It should be noted, however, that by reducing the size of the hydrogels, we may reduce the mechanical strength and stability, which are necessary for a microfluidic flow controller [16,27]; therefore, careful consideration has to be taken to ensure an optimum design.

**Figure 7:**
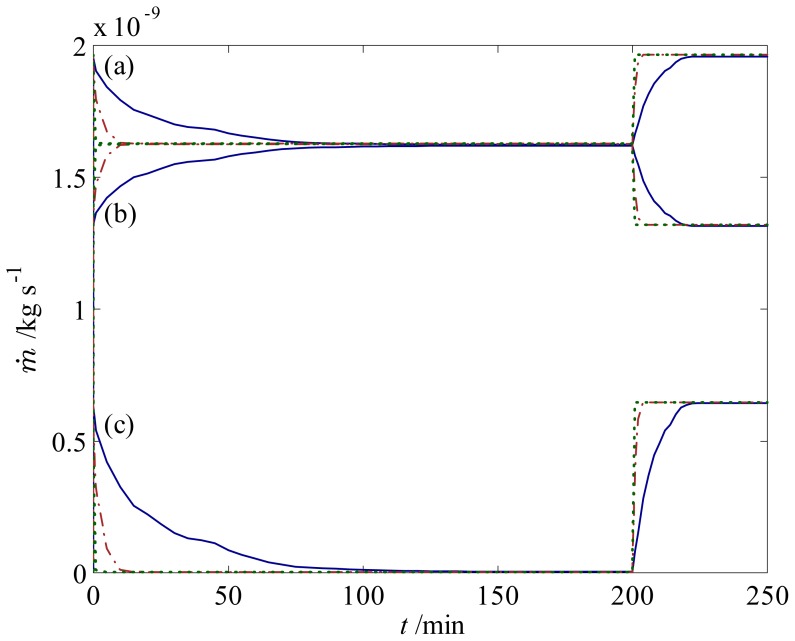
Response of the fluid flow during swelling and shrinking in a T-junction with one 300 *μ*m hydrogel which has ( 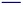) a base-case permeability; ( 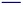), 10 times higher permeability, and ( 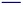) 100 times higher permeability. Mass flow rates of the fluid are for pH changes between 3 and 7 at (**a**) the inlet; (**b**) the left outlet; and (**c**) the right outlet.

The second strategy is achieved by implementing a 300 *μ*m hydrogel with 10 and 100 times higher permeability than the base-case hydrogel, which can be realized by utilizing macroporous hydrogels; see, e.g., [53,54]. With this approach, we find that the response times are around 10 and 95 times faster compared to the flow control system with a lower permeability, as presented in Figure 7. The faster response can intuitively be explained by the fact that swelling and shrinking kinetics mainly depend on the permeability of the hydrogel: on one hand, a low permeability induces a high resistance to the penetrating fluid flowing into the hydrogels, which in turn result in slow deformation response; on the other hand, a hydrogel with a high permeability allows for easier fluid penetration [35]. The utilized macroporous hydrogels, however, should possess a sufficiently low permeability, because hydrogels with too high permeability might allow a non-negligible amount of fluid to flow through them, which would defeat the purpose of flow control.

### 4.3. Flow Behavior Inside a T-Junction with a Hydrogel in Each Branch

**Figure 8:**
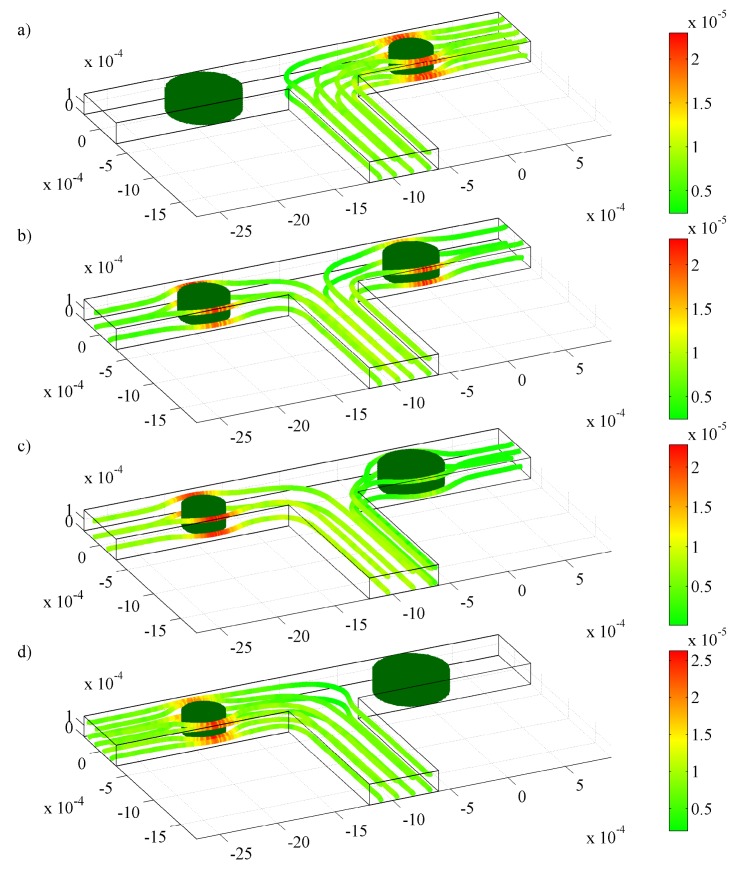
Streamlines and velocity distribution (*m s*^−1^) for laminar flow in a T-junction with two 300 *μ*m hydrogel for pH change between 3 and 7 at (**a**) *t* = 0 min; (**b**) *t* = 1 min; (**c**) *t*= 5 min; and (**d**) *t*= 25 min.

**Figure 9:**
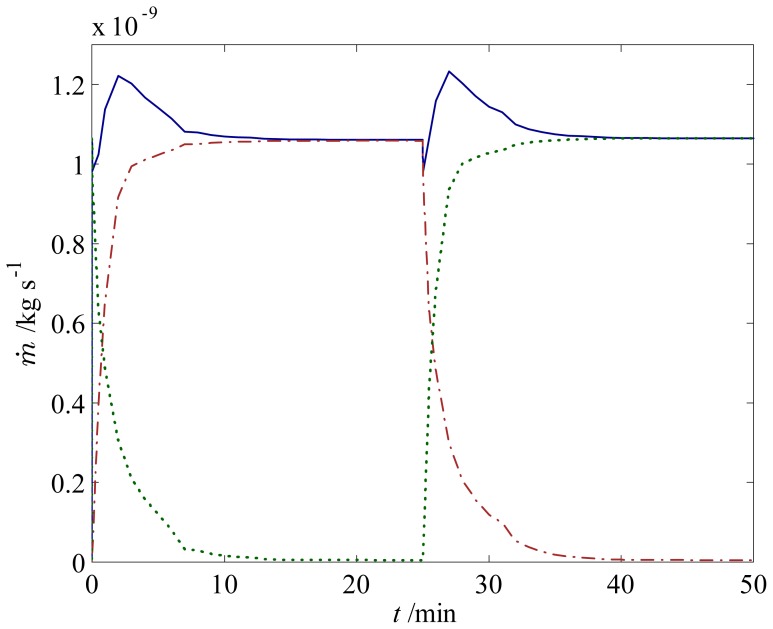
Response of the fluid flow during swelling and shrinking in a T-junction with two 300 *μ*m hydrogels in each branch. The mass flow rate of the fluid for pH changes between 3 and 7 are at ( 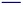) the inlet, ( 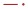) the left outlet, and ( 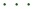) the right outlet.

In this configuration, two hydrogels with 10 times higher permeability than the base-case hydrogel with opposite behavior are introduced in the microchannel: a positive pH-responsive hydrogel, which swells as the pH increases, and a negative pH-responsive hydrogel, which shrinks as the pH increases. The fluid flow is thus either directed to the left or the right channel depending on the pH, as illustrated in Figure 8. Recalling that the shrinking is approximately ten times faster than the swelling for the conditions and HEMA hydrogel considered in this study, we expect that the switching between the positive and negative response hydrogels will not be symmetric. This is indeed the case, as during the first few minutes, the left channel starts to open before the right channel is fully closed. This, in turn, leads to fluid flow through both branches and causes an increase in the mass flow rate at the inlet, as shown in Figure 9. Clearly, one has to be careful when designing a flow sorter with hydrogels since the latter may still allow fluid flow through an undesired channel during the first few minutes (depending on response rate).

As the pH-positive hydrogel swells further, the right branch starts to be blocked; therefore, we see a decrease in mass flow rate at the inlet. When the positive pH-sensitive hydrogel reaches a new equilibrium (t ∼25 min), the entire right branch has been blocked, forcing all the fluid through the left branch (Figure 8d), for which we observe that the mass flow rate at the inlet is equal to that of the left branch. A step change in pH from 7 to 3 is then applied for which the positive pH-sensitive hydrogel shrinks whereas the negative pH-sensitive hydrogels swells towards the new equilibrium conditions.

In this configuration, each hydrogel plays the role of a sensor, regulator and an actuator for the fluid flow commonly handled by three different components. This particular configuration could be implemented in chemical or biochemical applications, where precise pH control is required; for example, in sequence determination of protein and DNA analysis [21].

## 5. Conclusions

A mathematical model for hydrogels embedded in a microfluidic T-junction that takes into account conservation of mass, momentum and ions for laminar, incompressible flow and for the sensing/deformation of a pH-sensitive hydrogel has been derived and presented. The predicted swelling behavior of a pH-sensitive hydrogel was validated with steady-state and transient experiments and achieved good agreement. The model was then employed to study the deformation behavior of hydrogels at various pH values and their impact on the fluid flow inside the microchannel where they act as autonomous valves. Two configurations were considered: a T-junction with hydrogels in one branch and a T-junction with hydrogels in each branch. Overall, the model could provide an insight into the swelling/shrinking behavior of hydrogels, which act as autonomous microvalves at various pH values.

From the numerical investigation, it was found that the response rate of hydrogels subject to pH changes is slow, which could defeat the flow control purposes. As such, two strategies to improve the response rate of the hydrogels were proposed and demonstrated: First, by using smaller hydrogels and, second, by employing hydrogels with higher permeability. It was found that the response rate improved 9 times when the hydrogel's size was reduced to 100 *μ*m from 300 *μ*m, and it could be further improved (up to 95 times) when macroporous hydrogels with 100 times higher permeability were implemented. It should be noted, however, that smaller hydrogels tend to have weaker mechanical strength while macroporous hydrogel may allow fluid to flow through them, which would defeat the purpose of flow control. Therefore, careful consideration is required when designing and synthesizing hydrogels for microfluidic flow control applications.

Finally, we would like to highlight that the model is not limited to pH-sensitive hydrogels; it can be extended to other stimuli-responsive hydrogels such as thermo-, electric-, alcohol-, and glucose-sensitive hydrogels.

## References

[1] Nguyen N.T., Wereley S.T. (2006). Fundamentals and Applications of Microfluidics.

[2] Jong J.D., Lammertink R.G.H., Wessling M. (2006). Membranes and microfluidics: A review. Lab Chip.

[3] Erickson D., Li D. (2004). Review: Integrated microfluidic devices. Anal. Chim. Acta.

[4] Koch M., Evans A., Brunnschweiler A. (2000). Microfluidic Technology and Applications.

[5] Delamarche E., Juncker D., Schmid H. (2005). Microfluidics for processing surfaces and miniaturizing biological assays. Adv. Mater..

[6] Squires T.M., Quake S.R. (2005). Microfluidics: Fluid physics at the nanoliter scale. Rev. Mod. Phys..

[7] Hossain S., Ansari M.A., Kim K.Y. (2009). Evaluation of the mixing performance of three passive micromixers. Chem. Eng. J..

[8] Ottino J.M., Wiggins S. (2004). Introduction: Mixing in microfluidics. Philos. Trans. R. Soc. London.

[9] Yi C., Li C.W., Ji S., Yang M. (2006). Microfluidics technology for manipulation and analysis of biological cells. Anal. Chim. Acta.

[10] Ohno K., Tachikawa K., Manz A. (2008). Microfluidics: Applications for analytical purposes in chemistry and biochemistry. Electrophoresis.

[11] Huh D., Gu W., Kamotani Y., Grotberg J.B., Takayama S. (2005). Microfluidics for flow cytometric analysis of cells and particles. Physiol. Meas..

[12] Beebe D.J., Mensing G.A., Walker G.M. (2002). Physics and applications of microfluidics in biology. Annu. Rev. Biomed. Eng..

[13] Chen Z., Wang J., Qian S., Bau H.H. (2005). Thermally-actuated, phase change flow control for microfluidic sytem. Lab Chip.

[14] Beebe D.J., Moore J.S., Bauer J.M., Yu Q., Liu R.H., Devados C., Jo B. (2000). Functional hydrogel structures for autonomous flow control inside microfluidic channels. Nature.

[15] Eddington D.T., Beebe D.J. (2004). Flow control with hydrogels. Adv. Drug Deliv. Rev..

[16] Qiu Y., Park K. (2001). Environment sensitive hydrogels for drug delivery. Adv. Drug Deliv. Rev..

[17] Roy I., Gupta N. (2003). Smart polymeric materials: Emerging biochemical applications. Chem. Biol..

[18] Liu Z.S., Swaddiwudhipong S., Cui F.S., Hong W., Suo Z., Zhang Y.W. (2011). Analytical solutions of polymer gel structures under buckling and wrinkle. Int. J. Appl. Mech..

[19] Marcombe R., Cai S., Hong W., Zhao X., Lapusta Y., Suo Z. (2010). A theory of constrained swelling of a pH-sensitive hydrogel. Soft Matter..

[20] Hong W., Liu Z.S., Suo S. (2009). Inhomogeneous swelling of a gel in equilibrium with a solvent mechanical load. Int. J. Solids Struct..

[21] Eddington D.T., Liu R.H., Moore J.S., Beebe D.J. (2001). An organic self-regulating microfluidic system. Lab Chip.

[22] Liu C., Park J.Y., Xu Y., Lee S.H. (2007). Arrayed pH-responsive microvalves controlled by multiphase laminar flow. J. Micromech. Microeng..

[23] Dong L., Jiang H. (2007). Autonomous microfluidics with stimuli-responsive hydrogels. Soft Mater..

[24] Park J.Y., Oh H.J., Kim D.J., Baek J.Y., Lee S.H. (2006). A polymeric microfluidic valve employing a pH-responsive hydrogel microspheres as an actuating source. J. Micromech. Microeng..

[25] Stoeber B., Yang Z., Liepmann D., Muller S.J. (2005). Flow control in microfluidic using thermally responsive triblock copolymers. J. Microelectromech. Syst..

[26] Wang J., Chen Z., Mauk M., Hong K.S., Li M., Yang S., Bau H.H. (2005). Self-actuated, thermo-responsive hydrogels valves for lab on chip. Biomed. Microdevices.

[27] Baldi A., Gu Y., Loftness P.E., Siegel R.A., Ziaie B. (2003). A hydrogel-actuated environmentally sensitive microvalve for active flow control. J. Microelectromech. Syst..

[28] Kurnia J.C., Birgersson E., Mujumdar A.S., Quah L.C. (2009). Mathematical modeling of hydrogels for microfluidic flow control. Adv. Mater. Res..

[29] Tehranirokh M., Majlis B.Y., Bais B. (2009). Design and simulation of a normally closed glucose sensitive hydrogel based microvalve. Microsyst. Technol..

[30] Ibrahim M.W.A., Saunders J.R., Wallied M. Hydrogel Microvalve Device Modeling and Simulation.

[31] De S.K., Aluru N.R., Jhonson B., Crone W.C., Beebe D.J., Moore J. (2002). Equilibrium swelling and kinetics of pH-responsive hydrogels: Models, experiments and simulations. J. Microelectromech. Syst..

[32] Li H., Yew Y.K., Ng T.Y., Lam K.Y. (2005). Meshless steady-state analysis of chemo-electro-mechanical coupling behavior of pH sensitive hydrogel in buffered solution. J. Electroanal. Chem..

[33] Li H., Yuan Z., Lam K.Y., Lee H.P., Chen J., Hanes J., Fu J. (2004). Model development and numerical simulation of electric-stimulus-responsive hydrogels subject to an externally applied electric field. Biosens. Bioelectron..

[34] Newman J., Thomas-Alyea K.E. (2004). Electrochemical Systems.

[35] Birgersson E., Li H., Wu S. (2008). Transient analysis of temperature-sensitive neutral hydrogels. J. Mech. Phys. Solids..

[36] Barry S.I., Holmes M. (2001). Asymptotic behavior of thin poroelastic layer. IMA. J. Appl. Math..

[37] Barry S.I., Mercer G.N. (1999). Flow and deformation in poroelasticity-I Unusual exact solutions. Math. Comput. Model.

[38] Sun D.N., Gu W.Y., Guo X.E., Lai W.M., Mow V.C. (1999). A mixed finite element formulation of triphasic mechano-electrochemical theory for charged, hydrated biological soft tissues. Int. J. Numer. Meth. Eng..

[39] Reddy J.N. (2008). An Introduction to Continuum Mechanics with Applications.

[40] Shirota H., Endo N., Horie K. (1998). Volume phase transition of polymer in water and heavy water. Chem. Phys..

[41] Hirotsu S. (1991). Softening of bulk modulus and negative Poisson's ratio near the volume phase transition of polymer gels. J. Chem. Phys..

[42] Oh K.S., Bae Y.C. (1998). Swelling behavior of submicron gel particles. J. Appl. Polym. Sci..

[43] Popov E.P. (1998). Engineering Mechanics of Solids.

[44] Solecki R., Conant R.J. (2003). Advanced Mechanics of Materials.

[45] Gu W.Y., Yao H., Huang C.Y., Cheung H.S. (2003). New insight into deformation-dependent hydraulic permeability of gels and cartilage, and dynamic behavior of agarose gels in confined compression. J. Biomech..

[46] Agarwal A.S., Landau U., Payer J.H. (2008). Modeling particulates effects on the cathode current capacity in crevice corrosion. J. Electrochem. Soc..

[47] Marcus Y. (1997). Ion Properties.

[48] Gawin D., Majorana C.E., Schrefter B.A. (1999). Numerical analysis of hygro-thermal behavior and damage of concrete at high temperature. Mech. Cohesive-Frict. Mater..

[49] Kaviany M. (2001). Principle of Heat Transfer.

[50] Gunasekaran S., Wang T., Chai C.X. (2006). Swelling of pH sensitive chitosan-poly(vinyl alcohol) hydrogels. J. Appl. Polym. Sci..

[51] Yew Y.K., Ng T.Y., Li H., Lam K.Y. (2007). Analysis of pH and electically controlled swelling of hydrogel-based micro-sensors/actuators. Biomed. Microdevices.

[52] De S.K., Aluru N.R. (2004). A chemo-electro-mechanical mathematical model for simulation of pH sensitive hydrogels. Mech. Mater..

[53] Zhao Q., Sun J., Zhou Q. (2007). Synthesis of macroporous poly(N-isopropylacrylamide) hydrogel with ultrarapid swelling-deswelling properties. J. Appl. Polym Sci..

[54] Zhang X.Z., Zhuo R.X. (2000). Novel synthesis of temperature sensitive poly(N-isopropylacrylamide) hydrogel with fast deswelling rate. Eur. Polym. J..

